# Diagnostic Accuracy of Periapical Radiography in Detection and Measurement of the External Root Resorption in Primary Molars: A Single-Blind Prospective Clinical Study

**DOI:** 10.1155/2022/7031086

**Published:** 2022-08-03

**Authors:** Shirin Taravati, Zohreh Balak, Vahid Rakhshan

**Affiliations:** ^1^Department of Pediatric Dentistry, School of Dentistry, Ahvaz Jundishapur University of Medical Sciences, Ahvaz, Iran; ^2^School of Dentistry, Ahvaz Jundishapur University of Medical Sciences, Ahvaz, Iran; ^3^Department of Anatomy, Dental School, Azad University of Medical Sciences, Tehran, Iran

## Abstract

**Introduction:**

Identifying the external root resorption plays an important role in treatment planning for deciduous teeth. Although proper accuracy of digital radiography in the diagnosis of external root resorption has been established in permanent dentition, it cannot be at all generalized to the primary root being superimposed by the succeeding permanent teeth. Interestingly, no study has assessed this in primary teeth yet. Thus, this study was undertaken for the first time.

**Methods:**

This was a single-blind prospective clinical diagnostic study performed on 501 observations (observed by 3 observers) pertaining to 167 roots of 45 maxillary/mandibular primary molars (in 6–8-year-old patients) which were indicated for extraction and did have succeeding permanent teeth. Digital parallel periapical radiographs were taken before dental extraction using a PSP sensor. Radiographs were printed and examined by two pediatric dentists. After the extraction, the extent of resorption was directly measured, twice, by third and fourth observers blinded to radiographic estimates. Radiographic errors and absolute errors (mm) were calculated. Various parameters were analyzed statistically.

**Results:**

Of the roots, 56.3% were really resorbed with (mean = 5.01 ± 2.10 mm). First/second observers failed to identify 19.6% and 38.3% of roots, respectively. Average errors were −0.77 ± 2.31 and 0.14 ± 2.19 mm in girls and boys, respectively (*P*=0.043, *t*-test). The factors “side, jaw, tooth type, and root type” did not affect errors (*P* > 0.05). Average absolute errors were 1.79 ± 1.47  mm (significantly above zero, *P* < 0.0005). No evaluated factor affected it (*P* > 0.1). Actual lesion sizes correlated with errors (Pearson *R* = 0.682, *P* < 0.0005) but not with absolute errors (*P*=0.464).

**Conclusion:**

Although many deciduous roots might be missed, digital radiography might still provide rather a good accuracy in diagnosis and measurement of external root resorption up to about 1.8 mm absolute error. Observers might overestimate resorption sizes in girls and in cases with actual lesions smaller than 3 mm. Size of the lesion can influence the direction of error (shortening/elongation), but not its directionless magnitude.

## 1. Introduction

The treatment pulpectomy is needed for teeth with evidence of chronic root pulp inflammation or necrosis. This treatment is contraindicated in teeth that have obvious loss of root structure or extensive internal or external resorption or whose infection has involved the permanent tooth bud [1, 2].

External root resorption in healthy deciduous teeth is part of the physiological process of replacing deciduous teeth with permanent teeth [1, 3]. Sometimes external root resorption may start pathologically due to factors such as inflammation caused by pulp infection, trauma, and orthodontic forces [3–5]. External inflammatory root resorption is a rapid process that is clinically characterized by increased tooth loosening, tenderness to percussion, and dull sound in the percussion, often with fistulas or swelling in the surrounding gingiva [3]. Radiographically, the periodontal space is enlarged, and the root surface becomes uneven [3, 6]. Pulpectomy can be done in teeth without extensive root resorption; therefore, the presence or absence of root resorption is one of the determining factors in the treatment planning for deciduous teeth [3].

Clinical diagnosis and treatment of root resorption is a difficult task [3]. Root resorption may begin at the internal or external surfaces of the root. Nevertheless, because conventional radiographs are two-dimensional, they are not entirely accurate in diagnosing root resorption [3]. When root resorption is not diagnosed and exacerbates, treatment prognosis becomes poorer. Therefore, radiographic detection of external root resorption is important to clinicians [7]. The common method for detecting external root resorption is conventional or digital intraoral radiography [2, 8–10]. This is also the method of choice for determining the length of external root resorption [11, 12]. However, geometric changes related to the vertical angle can shorten or lengthen the size of the image, resulting in errors [11, 13].

One of the problems while working on deciduous teeth is the diagnosis of root resorption. Three important factors are some major issues that make it difficult to identify roots on radiographs in children (i.e., the superimposition of the bone marrow space, especially the superimposition of the developing permanent tooth buds, and the normal pattern of physiological resorption of deciduous teeth) [2].

The available studies on permanent dentition have shown that root resorption is not completely visible on radiography [14–16]. As mentioned above, the identification of factors contributing to deciduous root resorption helps dentists to make more accurate diagnoses and prognoses and choose better treatment plans. This is because, in the presence of external root resorption in deciduous teeth, the prognosis of treatment decreases, and the success of treatment is negatively affected [3]. In addition, if the resorption is severe, the tooth must be extracted, and sometimes the diagnosis of root resorption helps the pediatric dentist decide between tooth extraction and keeping the tooth [17].

In the permanent dentition, the accuracy of digital radiography in the detection of root resorption has been assessed [3, 7, 8, 12]. Nevertheless, these results cannot be generalized at all to the primary dentition. This is because the radiographic conditions largely differ for the primary dentition due to the reasons stated above. Interestingly, the accuracy of digital radiography has never been examined in the primary dentition. Therefore, given the above-mentioned fact that the root resorption of primary teeth is a critical factor in diagnosis and treatment planning, we aimed to conduct this study.

## 2. Materials and Methods

This was a single-blind prospective clinical diagnostic study performed on 501 observations (data points) pertaining to 167 roots of 45 maxillary and mandibular primary molars in 45 patients observed by 3 observers.

The sample size was predetermined as 31, based on the parameters taken from the study of Lima et al. [3] (*P*=0.87, *d* = 0.12). It was augmented to 167 roots in 45 teeth of 45 patients to improve the precision.

The inclusion criteria were the primary first molars that were indicated for extraction owing to abscesses or root resorptions without any possibility of pulpectomy, looseness, and orthodontic treatment. The exclusion criteria comprised teeth with periodontal diseases, teeth with a history of a pulpectomy, teeth with natural physiological root resorption, children aged 3 to 10 years, patients with any systemic disease, a lack of the substituting permanent tooth bud, malformed teeth, and initially included teeth that later became fractured or broken at the time of extraction. All parents signed written consent forms after a thorough explanation of the study. No patients were exposed to any dose of X-rays for the sake of this research; all the radiographs were taken solely for treatment purposes. The ethics of this study were approved by the research committee of the university in accordance with the Helsinki declaration (ethics code: IR.AJUMS.REC.1397.231).

The primary molars in the upper and lower jaws of children aged 3–10 years (as an inclusion criterion) were selected according to the above-mentioned inclusion and exclusion criteria. Prior to the extraction of the selected teeth, periapical radiographs were taken by an experienced radiologist using a PSP sensor (Soredex, Tuusula, Finland) by the parallel technique using a film holder (Acteon Imaging, De Gotzen S.r.l., Varese, Italy, 70 kvp, 0.125 s, 200 mA/s, size 22 × 35 mm, ISO 0, speed E). The quality of radiographs was controlled by the radiologist, and in the case of low-quality imaging, it would be repeated. Only high-quality radiographs would be sent to the pediatric dentists (the observers). The radiographs had 508 PPI resolutions at a spot size of 50 *μ*m. They were printed (Drystar Axys, AGFA, Mortsel, Belgium) and placed in a special dark frame in a well-lit room. Each radiograph was examined by two pediatric dentists with at least 3 years of experience, working in a well-lit environment with dark frames. The extent of root resorption was evaluated and recorded in millimeters.

Afterward, the teeth were extracted and kept in a special container in 0.5% chloramine-T solution. The access cavity of the tooth was prepared using a diamond fissure bur (Tizkavan, Iran) attached to a high-speed handpiece by a pediatric dentist; then, endodontic files nos. 10 and 15 (K files, MANI, Japan) were inserted into the root canals. The root length and root resorption rates were determined using direct observation of the endodontic files, twice, by third and fourth experienced observers who were blinded to the results of digital radiographs. For this purpose, the counterpart nonresorbed roots of the same tooth and/or the contralateral tooth would be considered as the reference for the length of the nonresorbed root. The difference between this reference and the length of the resorbed root (measured using endodontic files) would be considered the actual resorption rate. The average of measurements done by both observers was considered the main extent of actual resorption.

### 2.1. Interobserver Agreement

The measurements recorded by different experts on digital radiographs and the teeth directly were entered as continuous (mm) into a spreadsheet. The interobserver agreement between the two radiograph assessors in terms of “identifying or failing to observe a given root on the radiograph” was calculated during the study using the Cohen Kappa. The interobserver agreements between the radiographic root resorption extents measured by the two radiograph assessors were computed during the study using the Cronbach Alpha. This was repeated for the errors.

### 2.2. Statistical Analyses

The data on radiographic estimations of each of the two observers were collected independently. The data pertaining to the actual errors observed directly by the third and fourth observers were averaged into one dataset; that is, for each root, the rounded average of the root resorption measured directly by the two observers was calculated and recorded. This was the gold standard.

The errors in the radiographic estimation of resorption extents were calculated for each root per observer as the *actual* resorption extent (the gold standard measured macroscopically) minus the radiographic resorption extent estimated by each radiograph observer. This way, negative and positive errors would indicate elongated and shortened radiographic root resorption estimates, respectively. Note that there were 2 different sets of errors (each for the difference between the actual resorption and the estimated radiographic resorption by one of the two radiograph evaluators).

Furthermore, absolute errors were calculated as the extent of the difference between the real resorption extent and the radiographic resorption estimate, regardless of the elongation/shortening type of error. Again, there were 2 sets of absolute errors (for 2 radiograph examiners).

Descriptive statistics and 95% confidence intervals (CI) were calculated for the root resorption extents, errors, and absolute errors. The errors were compared with the constant value of 0 mm (i.e., no error) using a one-sample *t*-test. A repeated-measures analysis of variance (ANOVA) was used to compare the resorption extents measured by the 3 observers (on the real teeth versus on their radiographs). An independent-samples *t*-test and a one-way ANOVA were used to compare the errors in the following groups: in boys versus girls, in the left versus the right sides of the mouth, in the maxilla versus the mandible, between different tooth types, and across different root types. Also, the correlations among the amounts of root resorption estimated on periapical digital radiographs by observers and the amount of real root resorption measured on the samples were assessed using a Pearson correlation coefficient. These analyses were repeated for the absolute errors (which had no direction but only the resorption extent). The software in use was SPSS 25 (IBM, Armonk, NY, USA). The level of significance was set at 0.05.

## 3. Results

There were 45 patients, all being 6 years old, except a 7-year-old patient and an 8-year-old one. Of them, 22 were girls, and 23 were boys. There were 167 roots in the sample, including 131 (78.4%) mandibular roots and 34 maxillary ones, 97 (58.1%) right roots and 70 left roots, and 132 (79.0%) roots of the first molars and 35 (21.0%) roots of the second molars. Of them, 45 (26.9%), 32 (19.2%), 46 (27.5%), 32 (19.2%), and 12 (7.2%) were mesiobuccal, mesiolingual, distobuccal, distolingual, and palatal, respectively.

### 3.1. Actual Root Resorptions

The macroscopic examination of the teeth showed that, of the 167 roots, 94 (56.3%) had been actually resorbed, and 73 were intact. The mean actual resorption extent of the 167 roots was 2.82 ± 2.95 mm. For the 94 resorbed roots (excluding the intact ones), the mean actual resorption extent was 5.01 ± 2.10 mm (minimum: 1, Q1: 3, median: 5, Q3: 7, and maximum: 10).

### 3.2. Radiographic Estimates of Root Resorption

The first radiograph examiner observed 136 (81.4%) roots and failed to see the remaining 31 roots. The mean radiographic resorption extent of these 136 roots observed by the first observer was 2.95 ± 2.40 mm (Table 1). After excluding the cases without actual root resorptions, there remained 74 roots observed by the first observer; the mean radiographic resorption rate of these 74 roots was 4.07 ± 2.31 mm (minimum: 0, Q1: 2, median: 4, Q3: 6, and maximum: 7).

The second radiograph evaluator observed 103 (61.7%) roots and could not identify the remaining 64 roots. The mean radiographic resorption extent of these 103 roots observed by the second observer was 2.89 ± 2.11 mm (Table 1). After excluding the really intact roots, there remained 59 roots observed by the second radiograph evaluator, with a mean radiographic resorption rate of 3.73 ± 1.96 mm (minimum: 0, Q1: 2, median: 4, Q3: 5, and maximum: 7).

#### 3.2.1. Difference between Radiographic Estimates and Actual Resorption Extents

The repeated-measures ANOVA did not show a significant difference among the 3 root resorption extents measured by the 3 observers (2 on radiographs and 1 direct observation (the rounded average of two directly measuring observers 3 and 4), *n* = 103 roots, *P*=0.173).

#### 3.2.2. Correlations

There were significant positive, moderate correlations between the actual root resorption extent with the radiographic root resorption estimates of the first radiograph evaluator (*n* = 136, *R* = 0.634, *P* < 0.0005) and the second one (*n* = 103, *R* = 0.483, *P* < 0.0005). Additionally, there was a strong correlation between the two radiographic estimates (*n* = 103, *R* = 0.732, *P* < 0.0005).

### 3.3. Errors in Radiographic Estimates of Resorption

**Figure 1:**
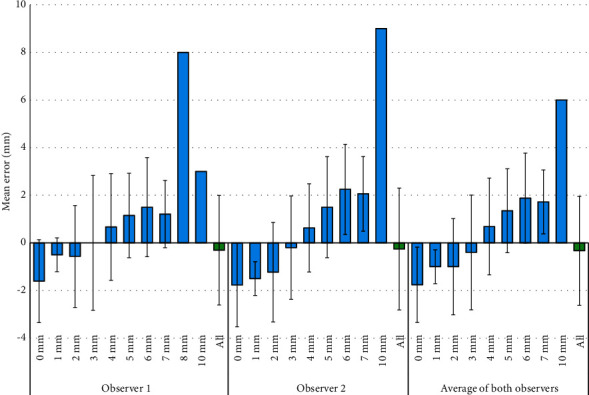
Mean (SD) of the errors of the radiograph examiners' radiographic root resorption estimates (mm) in the case of each of the actual resorption extents (mm). The errors are computed as the actual resorption extent minus the radiographic resorption estimate; therefore, negative and positive errors, respectively, point to elongated (overestimated) and shortened (underestimated) radiographic root resorption estimates.

After the exclusion of the 73 intact roots, the mean error of the first radiograph assessor was 0.78 ± 2.14 mm (*n* = 74, minimum: −5, Q1: 0, median: 0, Q3: 2, and maximum: 8), while the mean error of the second radiograph assessor was 0.86 ± 2.49 mm (*n* = 59, minimum: −5, Q1: −1, median: 1, Q3: 2, and maximum: 9), and the mean “average error” was 0.73 ± 2.16 mm for the two radiograph examiners combined (*n* = 59, minimum: −5, Q1: −0.5, median: 0.5, Q3: 2, and maximum: 6).  Figure 1 and Table 2 present the rest of the information regarding the radiographic resorption estimate errors.

#### 3.3.1. Error Directions

Taking into account the direction of the errors, the one-sample *t*-test showed that the mean errors of the first radiograph evaluator (*n* = 136, *P*=0.119), the second radiograph evaluator (*n* = 103, *P*=0.300), and their average (*n* = 103, *P*=0.141) were not significantly different from zero (Table 2).

#### 3.3.2. Role of Different Factors in Error Extents/Directions

According to the unpaired *t*-test and one-way ANOVA, there were no significant differences between the mean errors measured on the right versus the left sides, in the mandible versus the maxilla, in the first versus the second molars, and across different roots (*P* > 0.05, Table 3). However, the radiograph examiners tended to estimate slightly more positive resorption values in girls compared to boys (*P* < 0.05 for the second radiograph evaluator's error and the average error, Table 3).

#### 3.3.3. Correlations between Error Magnitudes/Directions and the Actual Sizes of Lesions

The Pearson correlation coefficient showed that there were positive, significant correlations between the actual sizes of the lesions with the average error magnitudes/directions of both radiograph evaluators (*n* = 103, *R* = 0.682, *P* < 0.0005), the error magnitudes/directions of the first radiograph evaluator (*n* = 136, *R* = 0.589, *P* < 0.0005), and the error magnitudes/directions of the second radiograph evaluator (*n* = 103, *R* = 0.693, *P* < 0.0005).

### 3.4. Directionless Absolute Errors of Radiographic Estimates

**Figure 2:**
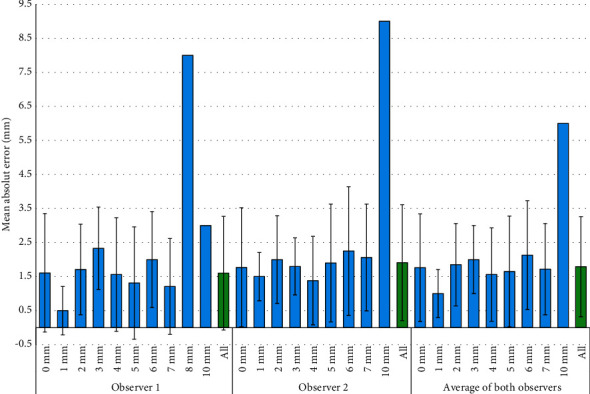
Mean (SD) of the directionless absolute error of resorption estimates (mm) in the case of each of the actual resorption extents (mm).

After the exclusion of the 73 intact roots, the mean absolute errors of the first and second radiograph evaluators were 1.60 ± 1.62 mm (*n* = 74, minimum: 0, Q1: 0, median: 1, Q3: 3, and maximum: 8) and 2.02 ± 1.68 mm (*n* = 59, minimum: 0, Q1: 1, median: 2, Q3: 3, and maximum: 9), respectively. The mean “average absolute error” was 1.81 ± 1.40 mm for both radiograph assessors combined (*n* = 59, minimum: 0, Q1: 0.5, median: 1.5, Q3: 3, and maximum: 6). The rest of the information on absolute errors of radiographic resorption estimates is presented in Figure 2 and Table 4.

#### 3.4.1. Comparison of Absolute Errors with Zero

The directionless absolute errors of the first radiograph examiner (*n* = 136, *P* < 0.0005), the second one (*n* = 103, *P* < 0.0005), and their average (*n* = 103, *P* < 0.0005, one-sample *t*-test) were significantly greater than zero (Table 4).

#### 3.4.2. Effects of Different Factors on Absolute Errors

None of the examined factors contributed to the extent of directionless absolute error (*P* > 0.05, unpaired *t*-test, Table 5).

#### 3.4.3. Correlations between Directionless Absolute Errors and the Actual Resorption Sizes

There were no significant correlations between the actual lesion sizes with the average absolute error of both radiograph examiners (*n* = 103, Pearson *R* = 0.073, *P*=0.464), the absolute errors of the first one (*n* = 136, *R* = 0.002, *P*=0.978), and the absolute errors of the second one (*n* = 103, *R* = 0.160, *P*=0.106).

### 3.5. Interobserver Agreements

The Cohen Kappa showed that there was a significant but only weak-to-moderate agreement between the two radiograph evaluators in terms of detecting (or “observing”) any given roots (Kappa = 0.537, *P* < 0.0005). The Cronbach Alpha showed a high interobserver agreement between the two radiograph examiners measuring the extents of radiographic root resorption on the radiographs (Alpha = 0.842, *P* < 0.0005). The interobserver agreement between the errors of the radiograph evaluators was very high (Alpha = 0.870, *P* < 0.0005). Regarding absolute errors, the interobserver agreement between the radiograph assessors was high (Alpha = 0.719, *P* < 0.0005).

## 4. Discussion

Radiographs can assist in the diagnosis of conditions such as caries, deep restorations near the pulp horn, success or failure of pulpotomy or pulpectomy, pulp alterations such as calcification and obstruction of the pulp, pathological root resorption, which can be inflammatory external or internal resorption, and diagnosis of periapical or interradicular radiolucency [2, 9–11]. At the time of treatment planning, the dentist should obtain a high-quality radiograph after the clinical examination. If the apical area cannot be clearly seen in this radiograph, a periapical radiograph of the involved tooth should be taken. Therefore, the dentist should be familiar with normal anatomical variations such as the large bone marrow space, superimposition of developing tooth buds, and the normal pattern of physiological resorption of deciduous teeth that can make radiographic diagnosis difficult in children [2, 18, 19]. Among the available radiography techniques, the accuracy of cone-beam computed tomography (CBCT) is higher than periapical radiography, and the accuracy of periapical radiography is higher than panoramic radiography [20]. However, due to the higher dose of radiation of CBCT [15] and the lower cost and easy access to periapical radiography, the latter was selected in this study. Besides, due to the limitations of the two-dimensional imaging in periapical radiography, we examined the diagnostic accuracy of this type of radiographic technique since the 3D CBCT method seemed to remain successful in the identification and measurement of root resorption in primary teeth.

Our results showed that the smallest errors/directions in the estimation of root resorption might happen when the actual root resorption is about 3 mm; if the actual resorption is greater than 3 mm, radiograph observers might tend to underestimate the extent of resorption. Nevertheless, regardless of the direction of errors, it was found that there is some absolute error associated with the radiographic estimation of the extent of resorption. Regardless of this 1.8 mm mean absolute error, our results agreed with many studies performed on permanent teeth, in which both digital and conventional radiographs have also been reliable in diagnosing root resorption [15, 16, 21, 22]. Nevertheless, in the study of Laux et al. [14], the rate of inflammatory resorption of the roots of permanent teeth was in fact very different from the rate of the resorption observed by us; in their study, 19% of teeth had radiographic resorption, while in reality, 81% of them had resorption. The reason for this difference could be in the type of radiographic technique used and the utilization of digital radiography in this study and the use of conventional radiography in the Laux study, 2004 [14].

Many biological factors have been claimed to contribute to the diagnostic accuracy of radiography at least in the permanent dentition; these include the size and location of resorptive lesions, anatomical differences in the jaws and teeth, and their mineral content [15]. Considering the factors influencing the diagnosis of root resorption, it seems that cancellous bone and less bone thickness may increase the ability to detect root resorption in deciduous teeth [2]. In general, experts may not be more discernment in estimating the rate of root resorption of the upper deciduous first molars, despite the anatomical differences and less superimposition of the roots in the maxillary molars. More studies are needed in this regard, as there is currently no similar study. Except for sex which could affect the direction of errors, no other factor could influence the direction of errors in the primary molars. Moreover, no factors, including sex, could affect the absolute errors in this study. These might be in part due to the much more complicated situation of the primary molars, which are obfuscated by the permanent successors.

Furthermore, technical factors, including the number and type of radiographs, the angle of the radiation tube, the exposure time, the sensitivity of the film, and the light where the radiographs were viewed, all affect the ability to detect the root resorption on radiography. It should be noted that, in this study, only periapical radiographs of the desired teeth were shown to radiograph evaluators. Moreover, the cases were not examined by panoramic radiography because it is not accurate enough to detect mild resorptions [15]. There might be some limitations in the diagnosis of root resorption, especially in the lingual roots of the mandibular molars, which, in the opinion of our study's observers, were not well visible. To solve this problem, two radiographs at two different angles can be used for better diagnosis. Nonetheless, due to the exposure of the child to more radiation and the poor cooperation of many children, it is difficult to prepare multiple radiographs for children. Also comparing new radiographs with old ones is one of the methods that may help to better diagnose root resorption [23].

One of the effective factors in diagnosing lesions is their size, in a way that the larger the size of the root resorption, the better its diagnosis. Many previous studies on the permanent dentition have shown that the larger the resorption, the greater the ability of experts to distinguish it [15, 21–25]. Although our findings considering the direction of the error agreed with this view, our absolute errors were not correlated with the size of the lesion. Since no earlier study exists on primary dentition, it is difficult to discuss this finding. After all, in the permanent dentition (researched before), the roots are not superimposed by succeeding tooth crowns, making them much clearer than the primary roots.

Another influencing factor in diagnosing root resorption is its location. Studies on permanent teeth have shown that resorption at the buccal and lingual surfaces of the root is not as visible as those on the mesial and distal surfaces [7, 23, 26]. One of the differences between our results and other studies could be the pattern of root resorption in deciduous teeth, which occurs mostly on the inner surfaces of the roots and is better seen because it is located on the proximal surfaces [2].

This study was limited by some factors. Because the observer's clinical judgment could affect the results, radiographs were shown to two people. In many cases, there was not much difference between the two observers, although in some cases there was a considerable difference. Root resorption has been seen on electron microscopic examination of teeth with healthy pulp and periodontal status. Therefore, root resorption is probably a normal physiological phenomenon that is exacerbated by pulp and periodontal problems. According to Bender [27], radiographic findings do not always indicate normal or pathological conditions. Also, radiographic lesions are smaller than their actual morphological size [27]. Histological examinations showed that root resorption occurs in various forms that are not accurately visible on radiography [14]. Therefore, the gold standard in the diagnosis of resorptive lesions is histological examination. And since the resorptive lesions are treated as soon as they are seen on the radiograph, early diagnosis improves the treatment [14]. Therefore, comparing the accuracy of periapical radiography and histological examination of roots in deciduous teeth is suggested in future studies. Moreover, the X-ray hazard disallowed us from conducting a randomized trial. Future studies should compare different estimation methods within the ethical limits.

## 5. Conclusions

Periapical radiography may provide rather useful information about the existence and extent of external root resorption in the primary molars with high reliability, of course noting the rather strong tendency of this technique to hinder the observers from detecting many existing roots on radiographs. The absolute estimates of resorption extents, regardless of the elongation/shortening direction, might be about 1.8 mm smaller or larger than the actual lesion. Overall, there was no considerable inclination towards more elongation or more shortening errors in the whole sample: elongation errors tend to occur more in the cases with less than 3 mm actual root resorption, while in roots with lesions larger than 3 mm, shortening errors tend to happen more. The direction of error (elongation/shortening) can be affected by the patient's sex only: observers might have some inclination to overestimate resorption sizes in girls. Regardless of error directions, none of the factors evaluated in this study may affect the absolute and directionless extent of error in radiographic estimates of the size of the lesions. Likewise, the extent of absolute error (regardless of elongation/shortening) might not be affected by the size of the actual resorptive lesion in the primary molars. As clinical implications, in primary dentition, periapical radiographs can provide useful information in detecting and measuring root resorption, although with about an average of 1.8 mm error in the size measurement and noting that periapical radiographs may hinder the detection of many primary roots.

## Figures and Tables

**Table 1:** Descriptive statistics and 95% CIs for the extents of radiographic resorption estimates (mm) observed by each of the radiograph examiners in the case of each of the extents of actual resorption (mm).

Observer	AR (mm)	*N*	Mean	SD	95% CI		Min	Max
Observer 1	0 mm	62	1.61	1.74	1.17	2.05	0	5
	1 mm	2	1.50	0.71			1	2
	2 mm	14	2.57	2.14	1.34	3.81	0	7
	3 mm	6	3.00	2.83	0.03	5.97	0	7
	4 mm	9	3.33	2.24	1.61	5.05	0	7
	5 mm	13	3.85	1.77	2.78	4.92	1	6
	6 mm	4	4.50	2.08	1.19	7.81	2	7
	7 mm	24	5.79	1.41	5.19	6.39	3	7
	10 mm	1	7.00					
	Total	136	2.95	2.40	2.54	3.36	0	7

Observer 2	0 mm	44	1.77	1.75	1.24	2.30	0	6
	1 mm	2	2.50	0.71			2	3
	2 mm	13	3.23	2.09	1.97	4.49	0	7
	3 mm	5	3.20	2.17	0.51	5.89	0	5
	4 mm	8	3.38	1.85	1.83	4.92	0	5
	5 mm	10	3.50	2.12	1.98	5.02	0	6
	6 mm	4	3.75	1.89	0.74	6.76	1	5
	7 mm	16	4.94	1.57	4.10	5.77	2	7
	10 mm	1	1.00					
	Total	103	2.89	2.10	2.48	3.30	0	7

AR, actual resorption; SD, standard deviation; CI, confidence intervals; min, minimum; max, maximum.

**Table 2:** Descriptive statistics and 95% CIs for the extents of error (mm, calculated as the actual resorption extent minus the radiographic resorption estimate) observed by each of the radiograph evaluators in the case of each of the extents of actual resorption (mm).

Error (mm)	AR (mm)	*N*	Mean	SD	95% CI		Min	Max
Observer 1	0 mm	62	−1.61	1.74	−2.05	−1.17	−5.0	0.0
	1 mm	2	−0.50	0.71			−1.0	0.0
	2 mm	14	−0.57	2.14	−1.81	0.66	−5.0	2.0
	3 mm	6	0.00	2.83	−2.97	2.97	−4.0	3.0
	4 mm	9	0.67	2.24	−1.05	2.39	−3.0	4.0
	5 mm	13	1.15	1.77	0.08	2.22	−1.0	4.0
	6 mm	4	1.50	2.08	−1.81	4.81	−1.0	4.0
	7 mm	24	1.21	1.41	0.61	1.81	0.0	4.0
	8 mm	1	8.00					
	10 mm	1	3.00					
	Total	136	−0.31	2.30	−0.70	0.08	−5.0	8.0

Observer 2	0 mm	44	−1.77	1.75	−2.30	−1.24	−6.0	0.0
	1 mm	2	−1.50	0.71			−2.0	−1.0
	2 mm	13	−1.23	2.09	−2.49	0.03	−5.0	2.0
	3 mm	5	−0.20	2.17	−2.89	2.49	−2.0	3.0
	4 mm	8	0.63	1.85	−0.92	2.17	−1.0	4.0
	5 mm	10	1.50	2.12	−0.02	3.02	−1.0	5.0
	6 mm	4	2.25	1.89	−0.76	5.26	1.0	5.0
	7 mm	16	2.06	1.57	1.23	2.90	0.0	5.0
	10 mm	1	9.00					
	Total	103	−0.26	2.56	−0.76	0.24	−6.0	9.0

Average error of both observers	0 mm	44	−1.76	1.58	−2.24	−1.28	−5.5	0.0
	1 mm	2	−1.00	0.71			−1.5	−0.5
	2 mm	13	−1.00	2.02	−2.22	0.22	−5.0	2.0
	3 mm	5	−0.40	2.41	−3.39	2.59	−3.0	3.0
	4 mm	8	0.69	2.03	−1.01	2.39	−2.0	4.0
	5 mm	10	1.35	1.76	0.09	2.61	−0.5	4.0
	6 mm	4	1.88	1.89	−1.13	4.88	0.0	4.5
	7 mm	16	1.72	1.34	1.00	2.43	0.0	4.0
	10 mm	1	6.00					
	Total	103	−0.33	2.29	−0.78	0.11	−5.5	6.0

AR, actual resorption; SD, standard deviation; CI, confidence intervals; min, minimum; max, maximum. Negative errors indicate that the root resorption estimated on radiographs was overestimated and longer than the actual root resorption. Positive errors mean an underestimation of the resorption.

**Table 3:** Descriptive statistics and 95% CIs for the extents of error (mm, calculated as the actual resorption minus the radiographic resorption estimate) on different sides and in different jaws, teeth, and roots. The *P* values are calculated using the unpaired *t*-test and one-way ANOVA.

Variable	Error	Factor	*N*	Mean	SD	95% CI		Min	Max	*P*
Side	Observer 1	Right	79	−0.61	2.17	−1.09	−0.12	−5.0	8.0	0.074
		Left	57	0.11	2.42	−0.54	0.75	−5.0	4.0	
	Observer 2	Right	58	−0.55	2.44	−1.19	0.09	−5.0	9.0	0.193
		Left	45	0.11	2.67	−0.69	0.91	−6.0	5.0	
	Average error of both	Right	58	−0.62	2.08	−1.17	−0.07	−5.0	6.0	0.151
		Left	45	0.03	2.50	−0.72	0.79	−5.5	4.5	

Jaw	Observer 1	Maxilla	36	−0.61	2.60	−1.49	0.27	−5.0	4.0	0.359
		Mandible	100	−0.20	2.18	−0.63	0.23	−5.0	8.0	
	Observer 2	Maxilla	36	−0.69	2.62	−1.58	0.19	−6.0	5.0	0.210
		Mandible	67	−0.03	2.51	−0.64	0.58	−5.0	9.0	
	Average error of both	Maxilla	36	−0.65	2.39	−1.46	0.16	−5.5	4.5	0.304
		Mandible	67	−0.16	2.23	−0.71	0.38	−5.0	6.0	
Tooth	Observer 1	D	106	−0.31	2.37	−0.77	0.15	−5.0	8.0	0.981
		E	30	−0.30	2.05	−1.07	0.47	−5.0	3.0	
	Observer 2	D	83	−0.36	2.61	−0.93	0.21	−6.0	9.0	0.424
		E	20	0.15	2.35	−0.95	1.25	−5.0	5.0	
	Average error of both	D	83	−0.35	2.32	−0.86	0.16	−5.5	6.0	0.897
		E	20	−0.28	2.19	−1.30	0.75	−5.0	2.5	

Root	Observer 1	MB	45	−0.53	2.22	−1.20	0.13	−5.0	4.0	0.870
		ML	29	−0.07	2.42	−0.99	0.85	−4.0	8.0	
		DB	46	−0.28	2.39	−0.99	0.43	−5.0	4.0	
		DL	4	0.50	1.00	−1.09	2.09	0.0	2.0	
		Palatal	12	−0.42	2.43	−1.96	1.13	−4.0	4.0	
	Observer 2 (no ML or DL detected)	MB	45	−0.16	2.26	−0.83	0.52	−5.0	5.0	0.711
		DB	46	−0.22	2.95	−1.09	0.66	−6.0	9.0	
		Palatal	12	−0.83	2.04	−2.13	0.46	−5.0	2.0	
	Average error of both	MB	45	−0.34	2.06	−0.96	0.27	−5.0	4.0	0.882
		DB	46	−0.25	2.57	−1.01	0.51	−5.5	6.0	
		Palatal	12	−0.63	2.12	−1.97	0.72	−4.5	3.0	

Sex	Observer 1	Female	69	−0.65	2.31	−1.21	−0.10	−5.0	4.0	0.077
		Male	67	0.04	2.25	−0.50	0.59	−5.0	8.0	
	Observer 2	Female	54	−0.83	2.52	−1.52	−0.15	−6.0	9.0	0.016
		Male	49	0.37	2.47	−0.34	1.08	−5.0	5.0	
	Average error of both	Female	54	−0.77	2.31	−1.40	−0.14	−5.5	6.0	0.043
		Male	49	0.14	2.19	−0.49	0.77	−4.5	4.5	

SD, standard deviation; CI, confidence intervals; min, minimum; max, maximum; D, the first molar; E, the second molar; MB, mesiobuccal; ML, mesiolingual; DB, distobuccal; DL, distolingual. Negative and positive errors would indicate elongated (overestimated) and shortened (underestimated) radiographic root resorption estimates, respectively.

**Table 4:** Descriptive statistics and 95% CIs for the extents of the directionless absolute error (mm) observed by each of the radiograph assessors in the case of each of the actual resorption extents (mm).

Absolute error (mm)	AR (mm)	*N*	Mean	SD	95% CI		Min	Max
Observer 1	**0**	62	1.61	1.74	1.17	2.05	0.0	5.0
	**1**	2	0.50	0.71	−5.85	6.85	0.0	1.0
	**2**	14	1.71	1.33	0.95	2.48	0.0	5.0
	**3**	6	2.33	1.21	1.06	3.60	1.0	4.0
	**4**	9	1.56	1.67	0.27	2.84	0.0	4.0
	**5**	13	1.31	1.65	0.31	2.31	0.0	4.0
	**6**	4	2.00	1.41	−0.25	4.25	1.0	4.0
	**7**	24	1.21	1.41	0.61	1.81	0.0	4.0
	**8**	1	8.00					
	**10**	1	3.00					
	Total	136	1.60	1.67	1.32	1.89	0.0	8.0

Observer 2	**0**	44	1.77	1.75	1.24	2.30	0.0	6.0
	**1**	2	1.50	0.71	−4.85	7.85	1.0	2.0
	**2**	13	2.00	1.29	1.22	2.78	0.0	5.0
	**3**	5	1.80	0.84	0.76	2.84	1.0	3.0
	**4**	8	1.38	1.30	0.29	2.46	0.0	4.0
	**5**	10	1.90	1.73	0.66	3.14	0.0	5.0
	**6**	4	2.25	1.89	−0.76	5.26	1.0	5.0
	**7**	16	2.06	1.57	1.23	2.90	0.0	5.0
	**10**	1	9.00					
	Total	103	1.91	1.70	1.58	2.25	0.0	9.0

Average absolute error of both observers	**0**	44	1.76	1.58	1.28	2.24	0.0	5.5
	**1**	2	1.00	0.71	−5.35	7.35	0.5	1.5
	**2**	13	1.85	1.21	1.11	2.58	0.5	5.0
	**3**	5	2.00	1.00	0.76	3.24	1.0	3.0
	**4**	8	1.56	1.37	0.41	2.71	0.0	4.0
	**5**	10	1.65	1.63	0.48	2.82	0.0	4.0
	**6**	4	2.13	1.60	−0.42	4.67	1.0	4.5
	**7**	16	1.72	1.34	1.00	2.43	0.0	4.0
	**10**	1	6.00					
	Total	103	1.79	1.47	1.50	2.08	0.0	6.0

AR, actual resorption; SD, standard deviation; CI, confidence intervals; min, minimum; max, maximum.

**Table 5:** Descriptive statistics and 95% CIs for the extents of directionless absolute error (mm) on different sides and in different jaws, teeth, and roots. The *P* values are calculated using the unpaired *t*-test and one-way ANOVAs.

Variable	Absolute error	Factor	*N*	Mean	SD	95% CI		Min	Max	*P*
Side	Observer 1	Right	79	1.52	1.66	1.15	1.89	0.0	8.0	0.492
		Left	57	1.72	1.69	1.27	2.17	0.0	5.0	
	Observer 2	Right	58	1.83	1.70	1.38	2.27	0.0	9.0	0.568
		Left	45	2.02	1.73	1.50	2.54	0.0	6.0	
	Average of both	Right	58	1.64	1.42	1.27	2.01	0.0	6.0	0.232
		Left	45	1.99	1.54	1.53	2.45	0.0	5.5	

Jaw	Observer 1	Maxilla	36	1.94	1.80	1.33	2.55	0.0	5.0	0.153
		Mandible	100	1.48	1.61	1.16	1.80	0.0	8.0	
	Observer 2	Maxilla	36	2.14	1.62	1.59	2.69	0.0	6.0	0.326
		Mandible	67	1.79	1.75	1.37	2.22	0.0	9.0	
	Average of both	Maxilla	36	2.04	1.42	1.56	2.52	0.0	5.5	0.208
		Mandible	67	1.66	1.49	1.29	2.02	0.0	6.0	

Tooth	Observer 1	D	106	1.69	1.69	1.36	2.01	0.0	8.0	0.262
		E	30	1.30	1.60	0.70	1.90	0.0	5.0	
	Observer 2	D	83	1.98	1.72	1.60	2.35	0.0	9.0	0.445
		E	20	1.65	1.63	0.89	2.41	0.0	5.0	
	Average of both	D	83	1.82	1.50	1.49	2.15	0.0	6.0	0.696
		E	20	1.68	1.41	1.02	2.33	0.0	5.0	

Root	Observer 1	MB	45	1.51	1.70	1.00	2.02	0.0	5.0	0.599
		ML	29	1.52	1.86	0.81	2.23	0.0	8.0	
		DB	46	1.76	1.62	1.28	2.24	0.0	5.0	
		DL	4	0.50	1.00	−1.09	2.09	0.0	2.0	
		Palatal	12	1.92	1.44	1.00	2.83	0.0	4.0	
	Observer 2	MB	45	1.67	1.51	1.21	2.12	0.0	5.0	0.267
		DB	46	2.22	1.93	1.64	2.79	0.0	9.0	
		Palatal	12	1.67	1.37	0.80	2.54	0.0	5.0	
	Average of both	MB	45	1.59	1.39	1.17	2.01	0.0	5.0	0.436
		DB	46	1.99	1.61	1.51	2.47	0.0	6.0	
		Palatal	12	1.79	1.20	1.03	2.55	0.0	4.5	

Sex	Observer 1	Female	69	1.78	1.60	1.40	2.17	0.0	5.0	0.204
		Male	67	1.42	1.73	1.00	1.84	0.0	8.0	
	Observer 2	Female	54	1.98	1.74	1.51	2.46	0.0	9.0	0.669
		Male	49	1.84	1.68	1.36	2.32	0.0	5.0	
	Average of both	Female	54	1.88	1.53	1.46	2.30	0.0	6.0	0.525
		Male	49	1.69	1.41	1.29	2.10	0.0	4.5	

SD, standard deviation; CI, confidence intervals; min, minimum; max, maximum; D, the first molar; E, the second molar; MB, mesiobuccal; ML, mesiolingual; DB, distobuccal; DL, distolingual.

## Data Availability

The raw data are available from the corresponding author upon reasonable request.
